# An Informational–Entropic Approach to Exoplanet Characterization

**DOI:** 10.3390/e27040385

**Published:** 2025-04-04

**Authors:** Sara Vannah, Ian D. Stiehl, Marcelo Gleiser

**Affiliations:** 1 Atmospheric and Environmental Research, Inc., Lexington, MA 02421, USA; 2 Department of Physics and Astronomy, Dartmouth College, Hanover, NH 03755, USAmgleiser@dartmouth.edu (M.G.); 3 Department of Physical Sciences, Earth and Environment, University of Siena, 53100 Siena, Italy

**Keywords:** exoplanet atmospheres, information theory, astrobiology, statistical techniques, Earth-like planets

## Abstract

In the past, measures of the “Earth-likeness” of exoplanets have been qualitative, considering an abiotic Earth, or requiring discretionary choices of what parameters make a planet Earth-like. With the advent of high-resolution exoplanet spectroscopy, there is a growing need for a method of quantifying the Earth-likeness of a planet that addresses these issues while making use of the data available from modern telescope missions. In this work, we introduce an informational–entropic metric that makes use of the spectrum of an exoplanet to directly quantify how Earth-like the planet is. To illustrate our method, we generate simulated transmission spectra of a series of Earth-like and super-Earth exoplanets, as well as an exoJupiter and several gas giant exoplanets. As a proof of concept, we demonstrate the ability of the information metric to evaluate how similar a planet is to Earth, making it a powerful tool in the search for a candidate Earth 2.0.

## 1. Introduction

The search for Earth-like, potentially inhabited planets has long been a key driver of discovery in astronomy. Current and near-future spectral observations are ushering this search into a new era. In the past, limited resolution and spectral coverage meant that exoplanets—especially rocky, potentially Earth-like planets—were assessed by a combination of inferred quantities (such as mass, radius, and distance from host star) and computer modeling. However, future missions such as the Atmospheric Remote-sensing Infrared Exoplanet Large-survey (ARIEL) [1], Large Interferometer For Exoplanets (LIFE) [2], the Habitable Worlds Observatory (HWO) [3,4], Earth-2.0 [5], and the European Extremely Large Telescope (E-ELT) [6] are focused partially or wholly on the search for an Earth-analog planet and include high-resolution, wide-passband spectroscopy, as well as advancements in telescope technology such as adaptive optics and novel observational techniques. As such, they promise to revolutionize the characterization of Earth-like exoplanets, by providing, for the first time, observational information about the chemical composition and structure of planets’ atmospheres. In fact, spectroscopy has already begun to revolutionize exoplanet characterization: JWST observations have been used to identify carbon dioxide, water, sulfur dioxide, and sulfur monoxide in the atmosphere of gas giant WASP-39 b [7,8,9,10], water in the gas giant WASP-96 b [11], and carbon dioxide and methane in K2-18 b [12]. Assessments of the Earth-likeness of exoplanets should take this new, invaluable source of information into account. Furthermore, we will argue that an assessment of the Earth-likeness of an exoplanet should be holistic, including not just the intrinsic astronomical properties of the planet in isolation but the atmospheric impacts of the life it hosts, if any. As we will show, information theory can provide a quantitative metric to compare the different observed exoplanets and effectively assess their Earth-likeness.

The term “Earth-like” is used in two distinct ways in the exoplanet community: as a qualitative description of a planet, or as a quantitative metric. Qualitative definitions vary across the astronomical community. The term is used most commonly to group rocky worlds with radii $0.5R_{⊕}≲R_{p}≲1.5R_{⊕}$, where $R_{⊕}$ is Earth’s radius, and $R_{p}$ is the exoplanet’s. An extra condition that is sometimes applied is that an Earth-like world must be in the habitable zone of its host star. Although useful as a first step, these methods offer an incomplete description of what it truly means for a planet to be similar to a living world like Earth. The current quantitative approaches to quantify the Earth-likeness or habitability of an exoplanet can be categorized into two classes: metrics relying on a short list of parameters describing the planet and approaches that use machine learning to manage a longer list of parameters. We briefly review these approaches here.

The Earth Similarity Index (ESI) [13] and related expansions [14,15] are perhaps the most widely used of the short-list planetary comparison metrics, for example, in NASA’s Planetary Habitability Laboratory. These metrics compute a weighted sum of the difference between the radius of the planet, surface gravity, distance from host star, and surface temperature of the planet compared to those of Earth. Similar measures such as the Constant Elasticity Earth Similarity Approach (CEESA) [16] and Cobb–Douglas habitability production function (CD-HPF) [17] use alternative calculations with different parameters (planetary radius, density, surface temperature, escape velocity, and eccentricity for CEESA, and radius, density, escape velocity and surface temperature for CD-HPF). Additionally, several metrics have been proposed that use parameters describing the habitability of a planet, rather than the intrinsic Earth-likeness. The Planetary Habitability Index (PHI), for instance, is a complement to the ESI meant to assess the likelihood that a planet is actually capable of hosting life and depends on the presence of a stable substrate, the energy available (largely from sunlight and chemistry), a chemical makeup that can form the polymeric chemistry necessary for life, and the presence of liquids that can be used as a solvent [13]. Another measure, the Biological Complexity Index (BCI) considers the presence of a stable substrate, surface temperature, age of the planet, geological activity, and energy availability [18].

A second class of quantitative Earth-likeness metrics uses machine learning to incorporate a larger list of planetary parameters. Sarkar et al. [19] use an unsupervised learning approach to search for anomalies in exoplanet features. Depending on the dataset used, these features may include parameters related to the mass, temperature, and size of both the planet and the star, the distance between the two, or orbital parameters. Saha et al. [20] use a supervised approach to assess habitability.

While both the qualitative and quantitative methods allow for the categorization of Earth-like planets, most share a common limitation: they require a discretionary, a priori choice of what parameters make a planet “Earth-like”. ESI and related measures, for example, choose a short list of parameters to describe the Earth-likeness of a planet. This parametrization becomes especially problematic when searching for potentially habitable Earth-like planets due to the unsettled debate of what planets qualify as “habitable” [21,22,23]. We propose that an improved method to assess the Earth-likeness of exoplanets should be agnostic of which parameters describe the Earth-likeness of the planet.

Furthermore, the previous methods have assessed either the habitability *or* the Earth-likeness of a planet. We argue that these two traits are not separable. In fact, Earth itself was and continues to be measurably changed by the presence of life. The detection of these so-called biosignatures in an exoplanetary spectrum would be a strong indicator that the planet hosts life [24]. Since our proposed method uses the spectra of planets, the metric is sensitive to the presence (or absence) of life on the planet. Potential biosignatures include the vegetative red edge [24,25,26], metabolic by-products in out-of-equilibrium chemistry such as combinations of methane and ozone, or methane and O_2_. Many of these molecular features will be detectable with the upcoming telescope missions under certain circumstances (e.g., low clouds) [27,28,29,30]. This makes our method well timed as we enter an era of higher-precision spectroscopic exoplanet characterization.

In this work, we propose an information theory-based method designed to be applied to transit spectroscopy. Like the ESI and its expansions, our method is a quantitative metric. Like atmospheric retrievals, our method can identify the Earth-likeness of an exoplanet’s atmospheric composition. And like molecular species identification, our method is sensitive to the presence of biosignature gases. The method introduced here is a companion to that of Vannah et al. [31]. While the method introduced in that work uses the information content of exoplanet atmospheres as a function of wavelength to analyze specific biosignature gases and habitability indicators, here we develop a method which uses the information content of a wide bandpass of the spectrum to assess the Earth-likeness—including the possible presence of life—of a particular planetary spectrum.

This is the main goal of the informational measure we propose here. It can be used to search for different kinds of exoplanets—not just Earth-like ones—focusing on their spectral signature. Within our approach, an Earth-like planet would be one that has a spectral signature with informational content (as defined in Section 2) close to Earth’s. An Earth clone would be one with a spectral signature identical to Earth’s–and thus with identical informational content. Since a true Earth clone is not realistic—due to instrumental limits and because no two planets may be perfectly alike—the difference in the information content between two planets may never be zero. Rather, two planets are most similar when their difference in information content is minimized. Our method provides a holistic, quantitative measure of the Earth-likeness of an exoplanet that complements the data already known from combining transit and Doppler methods that can furnish the radius, mass, and distance from the host star.

By quantifying the information content in the exoplanetary spectrum, we are able to identify—for spectral resolution with sufficiently low noise—spectra similar to those of Earth: the smaller the difference in information content, the more similar the spectra and thus the more Earth-like the exoplanet. Since we are still in the early days of acquiring high-quality spectral data (we will specify what high quality means for our method), our goal here is to offer a proof of concept, using our method to compare simulated exoplanets to a simulated Earth spectrum. We show that our information measure efficiently differentiates between Earth-like and Jupiter-like planets. We also demonstrate that our method is sensitive to variations in physical parameters that affect a planet’s spectral signature.

Section 2 (Information Measure) describes the information theory metric we employ to differentiate planets. Section 3 (Data) describes the simulations used to obtain data for this analysis. In Section 4 (Results 1), we show how our method is affected by varying the physical parameters for a sample Jupiter-like planet. This demonstrates the ability of our method to differentiate between planet types and to identify changes in planetary features. In Section 5 (Results 2), we show the results for a series of simulations of observed gas giant and rocky exoplanets. We compare these exoplanets with the simulated spectra of Earth, Jupiter, and a hot Jupiter clone to show how our method is able to discriminate between types of planets with realistic, diverse variations in planetary features. In Section 6 (Discussion and Conclusions) we summarize our results and expand on how the method may be used to identify the biosignatures associated with inhabited planets. Finally, we present an error analysis in the Appendix A.

## 2. Information Measure

The application of an informational entropic method to transmission spectra was introduced in a companion paper, Vannah et al. [31], and is summarized here for reference. In this work, we quantify the dissimilarity between two planetary spectra as the difference between the information contents contained in the two spectra, or more precisely, the distance in the information space. We treat the spectra as probability distributions,

(1) $$p_{\nu }=\frac{D_{\nu }}{\sum_{\nu }D_{\nu }},$$

for $D_{\nu }$, the transit depth of the spectrum at a particular wavenumber, ν. This takes the form of a modal fraction, probability distributions introduced by Gleiser and Stamatopoulos [32] to quantify the configurational entropy of spatially localized configurations of scalar fields. The information content of a modal fraction can be quantified through its Shannon entropy [33] *H*, given by

(2) $$H=-\sum_{\nu }p_{\nu }log\left(p_{\nu }\right).$$

Similarly, the Shannon entropy of a planetary spectrum quantifies the syntactic (non-meaningful, as opposed to semantic) information content of the spectrum. The information required to discriminate between two spectra is given by the Jensen–Shannon Divergence [34],

(3) $$D_{\mathrm{JS}}\left(p||q\right)=\frac{1}{2}D_{\mathrm{KL}}\left(p||r\right)+\frac{1}{2}D_{\mathrm{KL}}\left(q||r\right),$$

for $r=\frac{1}{2}\left(p+q\right)$ and $D_{\mathrm{KL}}$, the Kullback–Leibler Divergence [35],

(4) $$D_{\mathrm{KL}}\left(p||q\right)=\sum_{\nu }p_{\nu }log\left(\frac{p_{\nu }}{q_{\nu }}\right).$$

For an Earth spectrum with modal fraction $p_{\nu }$, $D_{\mathrm{JS}}\left(p||q\right)$ is a metric representing the amount of information lost by replacing the Earth spectrum with an exoplanet spectrum with modal fraction, $q_{\nu }$. A smaller information loss indicates more similar spectra; a $D_{\mathrm{JS}}$ of zero indicates identical distributions. Higher $D_{\mathrm{JS}}$ represents more information loss (and therefore less similarity) between two spectra.

In log base 2, the information measured in $D_{\mathrm{JS}}$ is in bits. A bit is the amount of information contained in an event with probability $\frac{1}{2}$, such as flipping a coin. Each “event” (data point) in the spectral distributions *p* and *q* has a probability far smaller than $\frac{1}{2}$, so the information encoded in each data point is far less than one bit. In this work, we use the natural logarithm so that $D_{\mathrm{JS}}$ is given in units of nats. We emphasize that $D_{\mathrm{JS}}$ is a purely comparative, rather than absolute, measure, as the amount of information contained in a spectrum depends on the resolution and noise of the specific datasets. However, for a collection of spectra with similar noise and resolution, $D_{\mathrm{JS}}$ can be used to quantitatively compare and group planets with similar spectral features.

While there are simpler approaches to compare planetary spectra—for example, simply taking the difference $\left[p_{\nu }-q_{\nu }\right]$—our method is, critically, a distance metric. Distance metrics are commonly used in statistics to create a metric space where two points in the space (here, the two points correspond to two planets) have some quantifiable distance between them. Like a distance measure in regular geometry, metrics measure distances that are always positive (or zero, for the distance between a point and itself), symmetric (the distance in information space between, say, Earth and Mars, is the same as the distance between Mars and Earth), and obey the triangle inequality.

In contrast to previous methods of exoplanet characterization, $D_{\mathrm{JS}}$ requires no prior knowledge to interpret the spectrum. This is critical: biosignatures or signs of habitability may resemble Earth’s atmosphere in ways we cannot predict a priori. There may also be examples of “life as we don’t know it”, producing biomarkers dissimilar from those we may know to look for. Relying on our incomplete understanding of the diversity of scenarios that may host life could cause us to miss inhabited planets. $D_{\mathrm{JS}}$ provides a holistic, quantitative measure of the Earth-likeness of an exoplanet. Furthermore, $D_{\mathrm{JS}}$ scales in a predictable manner with noise. This calculation in shown in Appendix A.

We note that $D_{\mathrm{JS}}$ alone is not able to isolate biosignatures, as it measures the difference in information content of the full spectrum rather than individual absorption lines. (We can think of it as a global quantity obtained from a given spectrum, like the area between two points under a curve obtained from its integral.) The identification of biosignatures requires some form of input knowledge (e.g., specific compounds related to biotic activity), while $D_{\mathrm{JS}}$ requires none. In a companion paper [31], we showed how the $D_{\mathrm{JS}}$-density per wave number can be used to isolate specific biosignatures in planetary spectra with limited input knowledge (compare to, for instance, Equation (4) or Equation (14) in [36]). Together, these two uses of $D_{\mathrm{JS}}$ allow for both the identification of a Earth-like planets (this paper) and for a detailed characterization of an exoplanet’s atmospheric composition. The global $D_{\mathrm{JS}}$ studied here may be used to search for exoplanets with the potential to host life, while the $D_{\mathrm{JS}}$-density may identify which of those exoplanets may actually be inhabited.

## 3. Data

As a test of our measure, we demonstrate that $D_{\mathrm{JS}}$ can identify different classes of exoplanet simulations using only their spectra. To illustrate this point, we use the radiative transfer code Exo-Transmit (https://github.com/elizakempton/Exo_Transmit (accessed on 24 January 2022)) for our simulations; further details on the mechanics of the simulation may be found in Kempton et al. [37]. The simulations are governed by seven parameters: equilibrium temperature used for a temperature-pressure profile, atmospheric equation of state, planetary surface gravity, planetary radius, stellar radius, a parameter controlling the cloud top pressure, and a parameter controlling the strength of Rayleigh scattering. For simplicity, we use an isothermal temperature-pressure profile, as transmission spectra are principally absorption spectra and thus minimally sensitive to temperature gradients. The simulation includes CH_4_, CO_2_, CO, H_2_O, NH_3_, O_2_, O_3_, C_2_H_2_, C_2_H_4_, C_2_H_6_, H_2_CO, H_2_S, HCl, HCN, HF, MgH, N_2_, NO, NO_2_, OCS, OH, PH_3_, SH, SiH, SO_2_, TiO, VO, Na, and K. The simulation uses a spectral resolution $\frac{\Delta \lambda }{\lambda }=10^{3}$, and has a wavelength range from 0.3 μm to 30 μm. The simulated spectra used in this work are shown in Appendix B.

For all of our simulations, we choose to set a fixed value for the Rayleigh scattering parameter while allowing the code to calculate the cloud top pressure. We use the literature values of each of the parameters to simulate an Earth spectrum and a Jupiter spectrum, using equilibrium chemistry for simplicity. We create a series of Jupiter clones with each individual parameter varied in isolation to demonstrate their effects on $D_{\mathrm{JS}}$, shown in Section 4.

We also simulate realistic gas giants and rocky exoplanets to demonstrate the ability of $D_{\mathrm{JS}}$ to differentiate between planet types. For each exoplanet class, we create ten planet simulations: six from observed exoplanets using parameter values from the literature, and four artificial planets designed to explore the parameter space. The parameters used to create these exoplanets are shown in Table 1. For comparison with the exoplanets, we simulate a Jupiter spectrum, an Earth spectrum, and the spectrum of a Jupiter clone with the temperature raised to 1200 K. These results are shown in Section 5. While Exo-Transmit cannot be directly validated against Earth or Jupiter transmission spectra, similar forward models have been validated through satellite observations of Earth by comparison with infrared, near-infrared, and visible transit spectra (using solar occultations) and Earthshine measurements [38,39,40,41,42].

## 4. Results 1: Comparing Spectra by Changing Physical Parameters

**Figure 1:**
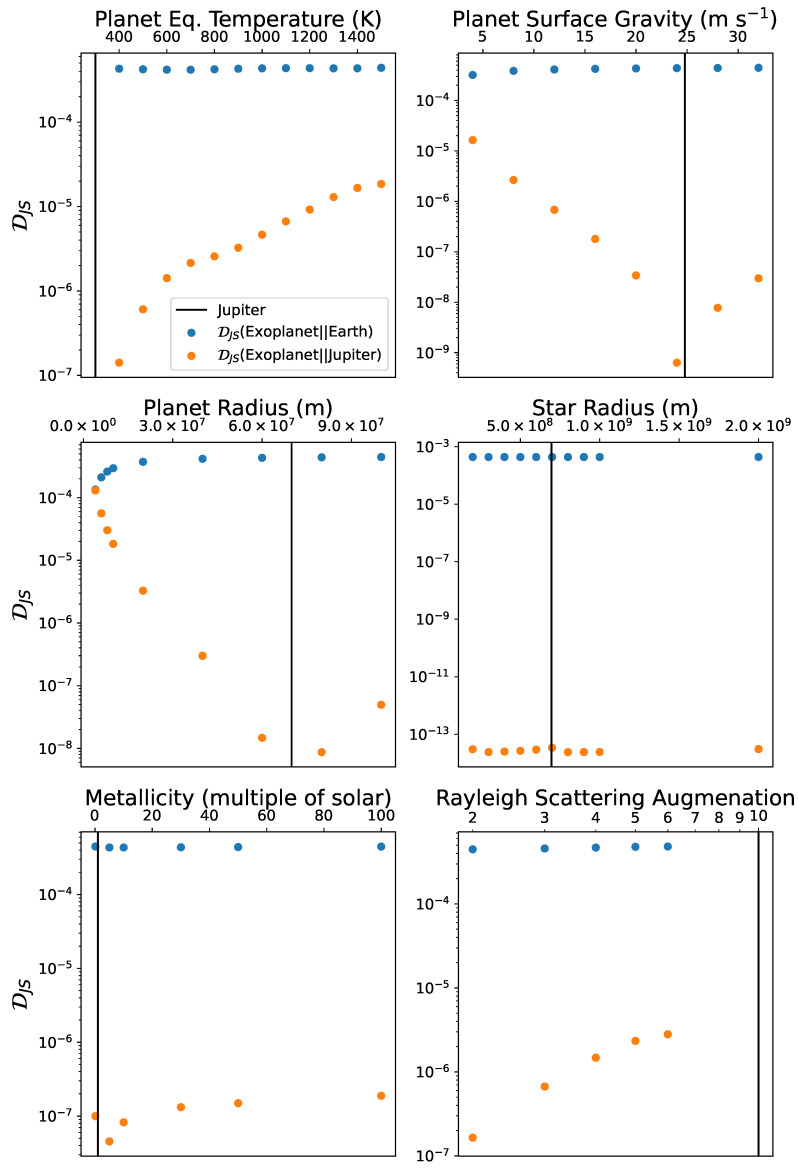
$D_{\mathrm{JS}}$ of a simulated, Jupiter-like exoplanet compared to Earth (blue dots) and to Jupiter (orange dots). Each plot represents the variation of one parameter. The vertical black line indicates the Jupiter value of the parameter.

We begin by testing our information measure in a simple scenario, varying the physical parameters of the simulated planets to assess how well our our informational measure picks up on these changes when compared to Earth and Jupiter. Figure 1 shows how varying each of the input parameters in isolation affects the $D_{\mathrm{JS}}$ of a model Jupiter-like planet relative to Earth and to Jupiter. Variations in the values of each of the planetary parameters away from their Jupiter values (vertical black line) incrementally increase the $D_{\mathrm{JS}}$ relative to Jupiter (orange points), displaying the growing dissimilarity between the two planets. This demonstrates that $D_{\mathrm{JS}}$ is sensitive to variations in planetary features. The $D_{\mathrm{JS}}$ relative to Earth (blue points) remains higher than the $D_{\mathrm{JS}}$ relative to Jupiter (orange points), confirming that small changes in the planetary parameters do not make the sample planet appear Earth-like.

Each of the plots tells a story illustrating the ability of $D_{\mathrm{JS}}$ to pick up on the underlying physics. Using the modal fraction ensures that the information content is not dependent on the continuum flux, only on the relative strength and shape of the absorption lines. For the equilibrium temperature, surface gravity, planet radius, and stellar radius—the top two rows of plots—varying the parameters varies the strength of the absorption lines in the modal fraction. For example, the scale height, *H*, of the planetary atmosphere is linearly dependent on the temperature, *T*, given by

(5) $$H=\frac{k_{B}}{\mu_{m}g}T,$$

for $k_{B}$ the Boltzmann constant, $\mu_{m}$ the mean molecular mass, and *g* the surface gravity on the planet. Increasing the scale height increases the size of the light-absorbing atmosphere. Therefore, the absorption line strength grows with temperature. As the strength of the exoplanet absorption lines diverges from the strength of the absorption lines in the Jupiter spectrum, the information contained in the two spectra also diverge. Vice versa, decreasing the temperature decreases the strength of the absorption lines. This results in a valley in the temperature plot (top left), where the lowest point in $D_{\mathrm{JS}}$—highest similarity between the two planets—sits nearest the Jupiter temperature (black line), while increasing incrementally as the temperature increases. Since, except for metallicity and Rayleigh scattering, changing the parameters away from the Jupiter value impacts the strength of the absorption lines, we see a valley occurring in nearly all plots. This illustrates the reliability of our approach to distinguish between different planetary properties. We note that the stellar radius plot in the middle right indicates that small variations in the stellar radius make little impact compared to variations in the other parameters. This is represented by the extremely low (of order $10^{-13}$) values of $D_{\mathrm{JS}}$ relative to Jupiter for the range of parameter values we analyzed.

Increasing the strength of Rayleigh scattering causes information divergence not by impacting the strength of the absorption lines but by creating a spectral tilt in the near-infrared [58]. This reduces $D_{\mathrm{JS}}$ at the low wavelength absorption lines in the spectrum. Therefore, just as with the other parameters, the values of the Rayleigh scattering factor closest to Jupiter’s have the lowest $D_{\mathrm{JS}}$. Similarly, changing the metallicity impacts the relative strength of absorption lines for different molecules. As a consequence, metallicities closest to Jupiter have the lowest $D_{\mathrm{JS}}$, although the differences are relatively small (smaller than an order of magnitude) compared to other physical parameters.

The second key result from the parameter variation analysis is that the values of $D_{\mathrm{JS}}$ comparing the modified Jupiters to Earth (blue dots) are consistently higher than those comparing to Jupiter. This indicates that our method is able to distinguish Earth-like and Jupiter-like planets for a wide range of parameters. The only exception occurs at the smallest planet radius in the middle left plot. As the planetary radius is decreased to even smaller values than the Earth radius, the $D_{\mathrm{JS}}$ compared to Jupiter and to Earth approach each other. This does not indicate that the planet is similar to Earth. Rather, it indicates that the distance in information space between the planet and Jupiter is similar to the distance between the planet and Earth, albeit in different directions. For the planet and Earth to be similar, the $D_{\mathrm{JS}}$ between the two would need to be small. How small would be hard to determine, unless we had a large sample of exoplanets that included ones that have spectra that are truly similar to Earth’s. We will be more specific about this when we discuss our results below.

## 5. Results 2: Differentiating Between Exoplanet Types with the Information Metric

In the previous section, we investigated the effects of changing a single physical parameter at a time as a first illustration of using $D_{\mathrm{JS}}$ as a discriminator of specific exoplanet properties when compared to a chosen baseline planet. Of course, in reality, the physical parameters that determine an exoplanet spectrum are often interdependent. Moving thus toward more concrete situations, in this section, we simulate the spectra of several observed rocky planets and gas giants to show that $D_{\mathrm{JS}}$ can indeed differentiate between planetary classes. We compare the two classes of planets to simulated Earth and Jupiter spectra, as well as to the spectrum of a Jupiter clone with the equilibrium temperature increased to 1200 K. Our results show that $D_{\mathrm{JS}}$ can identify which of the simulated worlds is closest to Jupiter or to Earth.

**Figure 2:**
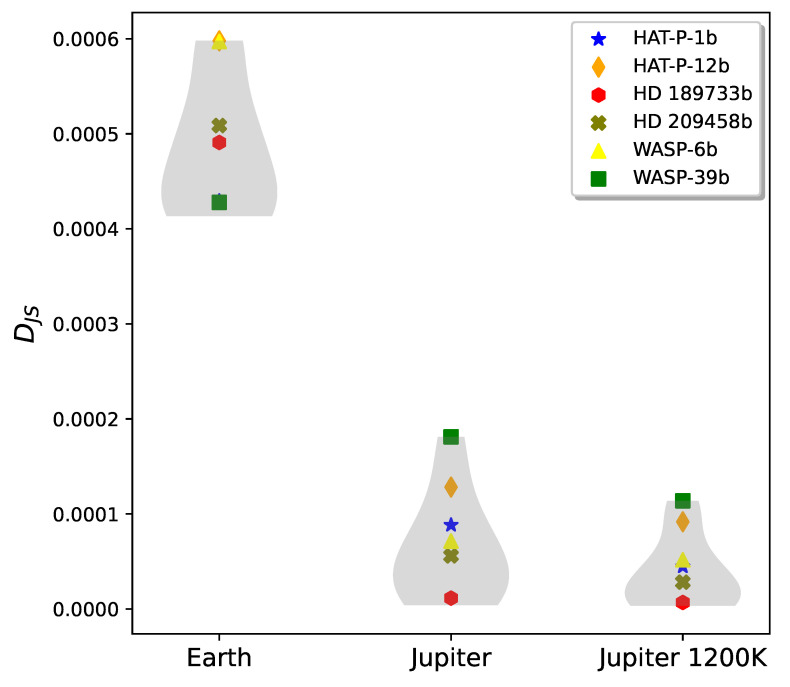
Violin plot showing the $D_{\mathrm{JS}}$ distributions comparing the simulated gas giants to Earth (**left**), to Jupiter (**center**), and to a Jupiter clone with the temperature raised to 1200 K (**right**). The width of the violin plot reads like a histogram, indicating the distribution of planets clustered around the mean $D_{\mathrm{JS}}$ value. The widest areas of the violin plot indicate the most common $D_{\mathrm{JS}}$ value for a particular distribution. The $D_{\mathrm{JS}}$ from six observed exoplanets are labeled with colored shapes; the remaining points in the $D_{\mathrm{JS}}$ distribution are from four exoplanet simulations. The physical parameters for the exoplanets are listed in Table 1.

In Figure 2, we find that the $D_{\mathrm{JS}}$ distribution comparing the six gas giants to the two Jupiter-like planets do not overlap with the $D_{\mathrm{JS}}$ distribution comparing them to Earth. This illustrates that $D_{\mathrm{JS}}$ is able to distinguish between Jupiter-like and Earth-like planets. There is, however, an overlap between the two distributions comparing the gas giants to Jupiter and to a 1200 K Jupiter. This is due to the similarity of the comparison planets. Still, the mean (bulge in the gray violin plots) of the 1200 K Jupiter $D_{\mathrm{JS}}$ distribution is the lower of the two distributions, indicating that $D_{\mathrm{JS}}$ is able to identify the temperature similarity of the hot Jupiters and the hotter, 1200 K Jupiter clone. We also show the results of specific comparisons for the six gas giants, each labeled by a different colored shape. For example, planet WASP-39b (identified by a green square) is clearly a hot Jupiter, most similar to the simulated Jupiter at 1200 K.

**Figure 3:**
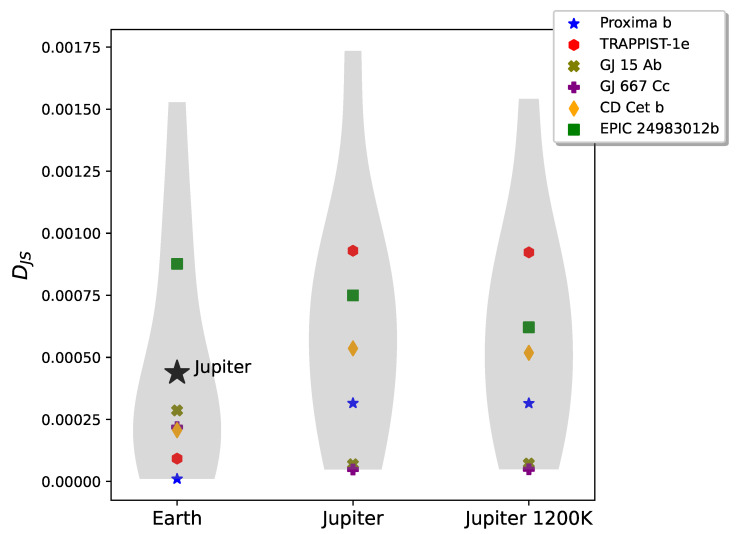
Violin plot showing the $D_{\mathrm{JS}}$ distributions comparing the simulated rocky planets to Earth (**left**), to Jupiter (**center**), and to a Jupiter clone with the temperature raised to 1200 K (**right**). The width of the violin plot reads like a histogram, indicating the distribution of planets clustered around the mean $D_{\mathrm{JS}}$ value. The $D_{\mathrm{JS}}$ from observed exoplanets are labeled with colored shapes; the remaining points in the $D_{\mathrm{JS}}$ distribution are from four exoplanet simulations. The physical parameters for the exoplanets are listed in Table 1. We include the $D_{\mathrm{JS}}$ comparing Jupiter and Earth in the leftmost violin plot for reference. Note that the position of Jupiter on this plot indicates that all the rocky planets but EPIC 24983012 are more Earth-like than Jupiter.

The rocky planet $D_{\mathrm{JS}}$ distributions in Figure 3 show more general overlap, although different exoplanets (colored shapes) are still clearly distinguished when compared to Earth (left) and to the two Jupiters (center and right). The mean $D_{\mathrm{JS}}$ (the bulge in each plot) comparing the rocky planets to Earth is lower than comparing them to Jupiter or to a 1200 K Jupiter, although the distributions are less distinct than those comparing gas giants in Figure 2. Still, when compared to Earth (left plot), with the exception of exoplanet EPIC 24983012b, all others have $D_{\mathrm{JS}}$ substantially lower than the gas giants of Figure 2, indicating that the method distinguishes between the two classes. In particular, we note how Proxima b (blue star) is the closest exoplanet to Earth in this sample. In contrast, EPIC 24983012b has an equilibrium temperature 1200 K hotter than Earth and a surface gravity more than twice that of Earth. The high $D_{\mathrm{JS}}$ between this exoplanet and Earth indicates that $D_{\mathrm{JS}}$ is able to pick up on these physical differences. EPIC 24983012b may be a super-Earth, but it is definitely not Earth-like. In general, super-Earths have larger radii and typically higher temperatures than Earth, moving them closer to the gas giants. Our results are consistent with this.

Within the accuracy of our results, we can now propose a preliminary criterion based on $D_{\mathrm{JS}}$ to distinguish between exoplanets. In Figure 2 and Figure 3, the leftmost violin plots depict comparisons with Earth: in Figure 2 between Earth and gas giants, and in Figure 3 between Earth and super-Earths or Earth-like worlds. We note that the results clearly depend on the sample of planets we are using to compare, and on the quality of the signal-to-noise ratio (see Appendix A). With those caveats, from Figure 2 and the comparison with Earth, if $D_{\mathrm{JS}}\left(\mathrm{exoplanet}||\mathrm{Earth}\right)>3\times 10^{-4}$, the exoplanet is most probably a gas giant. From Figure 3, the exoplanets that have spectra closest to Earth’s would have $D_{\mathrm{JS}}\left(\mathrm{exoplanet}||\mathrm{Earth}\right)\leq 2\times 10^{-4}$, which from our sample would include the two most Earth-like exoplanets, TRAPPIST-1e and Proxima b.

## 6. Discussion and Conclusions

In this work, we have introduced a new metric that makes use of modern spectroscopic data to assess the Earth-likeness of planetary spectra. The method can also be applied to compare a planetary spectra to another planet—say, Jupiter, as in our work—providing an agnostic measure based on any reference spectrum, given sufficient accuracy. Our metric has several key strengths: It is a quantitative assessment that can be used to rank the Earth-likeness of different exoplanets, placing it into a well-established field of indices assessing similarity to Earth. Unlike these indices, it does not require an a priori choice of what parameters make a planet Earth-like, making our method independent of the unsettled debate of what makes a planet Earth-like or habitable. Furthermore, the accuracy of our information metric for assessing the Earth-likeness of exoplanets will increase with data availability from future and upcoming high-resolution wide-passband spectroscopic missions. Finally, our quantitative method is holistic, as it does not separate Earth from its life.

This work is a companion to Vannah et al. [31], which introduced a wavelength-specific comparison (a $D_{\mathrm{JS}}$-wavelength density) between two spectra to search for biosignatures. The difference in information content between an Earth-as-an-exoplanet spectrum and the spectrum of an exoplanet at a wavelength corresponding to a potential biosignature gas can indicate the likelihood that the planet hosts Earth-like life. Similarly, as introduced in this work, the total information content relative to Earth contained in a spectral window can indicate how Earth-like an exoplanet is. While the method introduced in Vannah et al. [31] can be used to search for specific gasses potentially associated with biosignatures, the metric introduced here can be used to search for potentially habitable planets in a global “Earth-like” sense.

Stephens et al. [59] and other works cited therein show that $D_{\mathrm{JS}}$ is sensitive to patterns in the data. The better the signal-to-noise ratio and the wavelength resolution of the spectrum, the more efficient the method. This dependence is explored further in Appendix A. Similar approaches using the information entropic content of a spectrum or a field have been shown to be effective in a wide variety of astrophysical, cosmological, and high-energy physics scenarios: [32,36,59,60,61,62,63,64,65] is an incomplete list. In this work, we use $D_{\mathrm{JS}}$ similarly to identify syntactic (non-meaningful, or non-semantic) information similarities between planetary spectra.

Sandford et al. [66] adopt a similar strategy, demonstrating that patterns in the information of planetary systems can reveal the mass and radius of a missing planet without input physics. Similarly, we contend that the information content of exoplanet atmospheres as contained in its modal fraction and $D_{\mathrm{JS}}$ can help identify exoplanets with potential habitability without the need for prior knowledge of a planet’s physical properties. Indeed, with a large enough database, the information encoded in the spectrum will help elucidate some of these properties. (Assuming, of course, that the spectrum has sufficient resolution).

Similar complexity measures, pattern recognition, and information theory strategies have also been proposed for a limited selection of other astrobiology purposes. These include, amongst others, in situ complexity measures [67,68,69], time series information content of planetary reflectance spectra [70], information gain as a tool for determining the observational parameters necessary to observe biosignature gases [71], semantic (meaningful) information to describe exchange on information in daisy world models [72], detailed chemical networks as potential biosignatures [73], and network analysis to distinguish between biogenic and abiotic sources of atmospheric chemistry [74]. We especially highlight the related work in Guez and Claire [75], who found that a spectral clustering algorithm can characterize simulated JWST spectra (in particular, whether they are from oxidizing or reducing atmospheres and mixing ratios for CO_2_ and O_2_) agnostically of the molecular features in the spectrum.

Finally, we note a connection between our proposed spectral $D_{\mathrm{JS}}$ method and methods using the Gibbs free energy of a planetary atmosphere as a biosignature [26,76,77,78,79,80]. The Gibbs entropy of an ensemble is given by

(6) $$S_{Gibbs}=-k_{B}\sum_{i}p_{i}log\left(p_{i}\right)$$

for $p_{i}$ the *i*th member of a particular ensemble, and $k_{B}$ the Boltzmann constant. The key difference between this expression and the Shannon entropy expression (Equation (4)) is that the Gibbs entropy is a sum over molecular species *i*, while Shannon entropy in our metric is a sum over wavenumber ν. In an idealized sense, however, the strength (e.g., absorption) of the transmission spectrum at a given wavelength is correlated to the abundance of the molecule(s) that absorb at that wavelength. This means, in a sufficiently high-resolution and low-noise spectrum, that Shannon entropy and Gibbs entropy are correlated. The Gibbs free energy, *G*, of a system is

(7) $$\Delta G=\Delta H-T\Delta S_{Gibbs}$$

for *H* the enthalpy of the construction of a state, and *T* the absolute temperature. This relation between ΔG and $S_{Gibbs}$ implies a further correlation between our spectral information metric and Gibbs free energy as a potential biosignature. In contrast to the proposed methods to assess the Gibbs free energy of exoplanet atmospheres, however, our method does not require performing a retrieval to determine the abundance of a set of predetermined molecular species, $p_{i}$. Rather, it determines the information content from the spectrum itself. We plan to study the correlation between Gibbs free energy and our metric, especially its dependence on observational parameters, in future work.

In this paper, we demonstrate the efficacy of this method using simulated exoplanet data. As a proof of concept, we use these data to show that our method recovers the results we would expect. We first show that $D_{\mathrm{JS}}$ is sensitive to a wide variety of planetary parameters. This indicates that our information measure can be used to identify planetary features. This analysis also shows that $D_{\mathrm{JS}}$ is able to differentiate between planets with Earth-like and Jupiter-like spectral characteristics with reasonable variation in planetary features. To further validate this ability, we use simulations of observed exoplanets—including high-interest exoplanets such as habitable zone planets Proxima b and TRAPPIST-1e—to show that our method is able to distinguish planet types with realistic data. In both of these illustrations, $D_{\mathrm{JS}}$ is able to identify Earth-like and Jupiter-like planets, even without a priori knowledge of what spectral features differentiate the two planet types. This is important, as our limited understanding of the complex scenarios with the potential to host life could cause us to miss habitable planets. More detailed simulations—including those with non-equilibrium chemistry—will allow for more accurate classification with a wider class of planets.

We also note that we have not considered here the impact of stellar flares on TRAPPIST-1e, which may have a significant impact on its potential biosignature and even whether it may host an atmosphere [81,82]. The analysis in this work is meant to illustrate the ability of our method; a true determination of the similarity of TRAPPIST-1e to Earth will require more detailed simulations such as Lin and Kaltenegger [83] or Fauchez et al. [84].

While the $D_{\mathrm{JS}}$ distributions comparing rocky planets to Earth, to Jupiter, and to a warm Jupiter clone show overlap, we argue that most of the observed rocky planets lie at the low end of the $D_{\mathrm{JS}}$ distribution when compared to Earth but are more spread out when compared to Jupiter and 1200 K Jupiter. The overlap in $D_{\mathrm{JS}}$ can be greatly reduced by removing the four fabricated rocky planets simulations. The exception—the one observed exoplanet with a high $D_{\mathrm{JS}}$ relative to Earth—is EPIC 24983012b, a very large, hot planet. These physical parameters bring the spectrum of the exoplanet closer to a Jupiter or hot Jupiter-like planet than to Earth. The ability of $D_{\mathrm{JS}}$ to identify these physical differences in fact underscores our method—EPIC 24983012b is not Earth-like.

The previous observations of exoplanetary spectra use narrow bandpass filters, often with low resolution. As a result, the information content of these spectra is very low. Even with JWST, the shot noise limit of transmission spectroscopy limits the signal-to-noise ratio achievable without advanced spectroscopic methods proposed in near-future missions. In the Appendix A, we show how $D_{\mathrm{JS}}$ varies with a simple Gaussian noise model. In reality, noise in a transmission spectrum depends on a complex mix of instrument factors, planetary features (such as the distance between the planet and host star), and observational conditions (such as the number of observed transits). In future work, we hope to use spectra with simulated noise to test the dependence of our method on the signal-to-noise ratio of next generation telescope data. This can be performed for JWST using the JWST simulators JexoSim-2.0 [85] or PandExo [86], testing the efficacy of the $D_{\mathrm{JS}}$ method on JWST data. We note that JWST recently confirmed the mission’s ability to resolve spectral biosignatures with detections of carbon dioxide in the atmosphere of WASP-39b [87]; methane in WASP-80b [88]; water, carbon dioxide, sulfur dioxide, and sulfur monoxide in the gas giant WASP-39 b [7,8,9,10,87]; and methane and carbon dioxide in K2-18b [89,90]. We anticipate that the method will be most helpful in future missions designed specifically for spectroscopy of potentially Earth-like planets (identified by non-spectral parameters like their mass and orbital distance) such as HWO.

We note that, while our method is agnostic to the features that make a planet Earth-like—including life—it is not a direct measure of biosignatures. Our method assesses the similarity of an exoplanets spectrum to that of Earth. While life has a decisive impact on this similarity, a low $D_{\mathrm{JS}}$ does not, in-and-of-itself, indicate life. Research is actively being conducted into true so-called “agnostic biosignatures”. Refs. [67,68,69,70,73] is an incomplete list. These methods may be able to identify life without assuming the form that life takes, while our method may identify Earth-like planets without assuming what features define a planet’s Earth-likeness.

## Figures and Tables

**Table 1:** The parameters used to generate realistic exoplanet simulations. The gas giant simulations are shown in the top half of the table, while the rocky planets are shown in the bottom half. The two planetary classes are separated by a horizontal line. *^a^* Sources: Montañes-Rodriguez et al. [43], Sato and Hansen [44]; *^b^* Source: Liu et al. [45]; *^c^* Source: Hartman et al. [46]; *^d^* Source: Boyajian et al. [47]; *^e^* Source: del Burgo and Allende Prieto [48]; *^f^* Source: Gillon et al. [49]; *^g^* Source: Faedi et al. [50]; *^h^* Source: Kempton et al. [37]; *^i^* Sources: Lin and Kaltenegger [51], Barnes et al. [52]; *^j^* Sources: Lin and Kaltenegger [51], Delrez et al. [53]; *^k^* Source: Pinamonti et al. [54]; *^l^* Source: Anglada-Escudé et al. [55]; *^m^* Source: Bauer et al. [56]; *^n^* Source: Hidalgo et al. [57].

Name	Equilib. Temp. (K)	Equation of State	Surface Gravity (g)	Planet Radius (m)	Stellar Radius (m)	Rayleigh Scattering Factor
Jupiter *^a^*	300	1X	24.79	6.99 × 10^7^	6.96 × 10^8^	10
HAT-P-1b *^b^*	1300	5X, graphite rainout	7.5	9.44 × 10^7^	8.17 × 10^8^	10
HAT-P-12b *^c^*	1000	1X	5.6	6.86 × 10^7^	4.87 × 10^8^	200
HD 189733b *^d^*	1200	1X	21.4	8.15 × 10^7^	5.60 × 10^8^	500
HD 209458b *^e^*	1500	0.1X	9.4	9.72 × 10^7^	8.35 × 10^8^	10
WASP-6b *^f^*	1200	1X	8.7	8.72 × 10^7^	6.05 × 10^8^	1000
WASP-39b *^g^*	1100	1X	4.1	9.08 × 10^7^	6.23 × 10^8^	1
Gas giant 1	1400	5X, graphite rainout	12.8	8.45 × 10^7^	5.02 × 10^8^	1000
Gas giant 2	700	1X	20.1	6.27 × 10^7^	8.95 × 10^8^	100
Gas giant 3	1000	1X, 0.2 C/O ratio	8.8	9.23 × 10^7^	6.48 × 10^8^	10
Gas giant 4	1300	1X, 0.8 C/O ratio	17.2	6.90 × 10^7^	9.23 × 10^8^	1
Earth *^h^*	300	1X	9.8	6.37 × 10^6^	6.96 × 10^8^	1
Proxima b *^i^*	300	1X	10.9	6.82 × 10^6^	9.82 × 10^7^	1
TRAPPIST-1e *^j^*	300	1X	7.2	5.85 × 10^6^	8.15 × 10^7^	1
GJ 15 Ab *^k^*	300	0.1X	12.4	9.88 × 10^6^	2.85 × 10^8^	10
GJ 667 Cc *^l^*	300	0.1X	15.7	9.81 × 10^6^	2.92 × 10^8^	1
CD Cet b *^m^*	500	1X	11.7	1.16 × 10^7^	1.18 × 10^8^	0
EPIC 24983012b *^n^*	1500	1X	22.6	1.24 × 10^7^	1.19 × 10^9^	1
Rocky planet 1	700	1X	14.8	9.87 × 10^6^	8.80 × 10^8^	10
Rocky planet 2	400	5X	10.4	6.02 × 10^6^	4.53 × 10^8^	1
Rocky planet 3	300	1X	8.4	7.12 × 10^6^	9.25 × 10^8^	1000
Rocky planet 4	1000	10X	12.8	8.54 × 10^6^	1.02 × 10^9^	1

**Figure A1:**
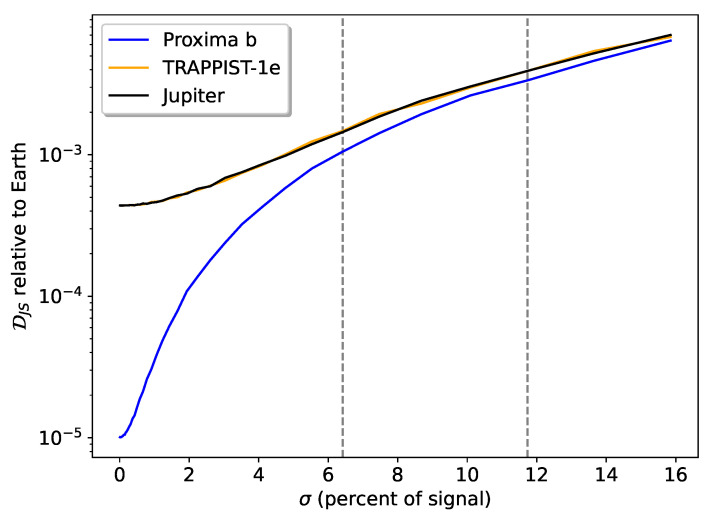
$D_{\mathrm{JS}}$ relative to Earth of two Earth-like exoplanets—Proxima b (blue line) and TRAPPIST-1e (orange line)—and Jupiter (black line) for different strengths of noise. The noise strength is changed by choosing σ, the width of a mean-zero Gaussian distribution from which the noise at each point in the transmission spectrum is drawn, to be a particular percentage of strength of the transmission spectrum for each planet. For example, σ of 20% means that a noisy transmission spectrum for Jupiter (and each of the four planets) has been created by adding noise at each point in the spectrum where the noise is drawn from a Gaussian distribution with a width set to be 20% of the mean transit depth of the Jupiter spectrum. Gray dashed lines show the values of σ when the two Earth-like planets are within 10% of each other (left) and when Proxima b and Jupiter are within 10% of each other. These were chosen as locations to quantify how small the noise must be in order for the planets to be distinguishable.

**Figure A2:**
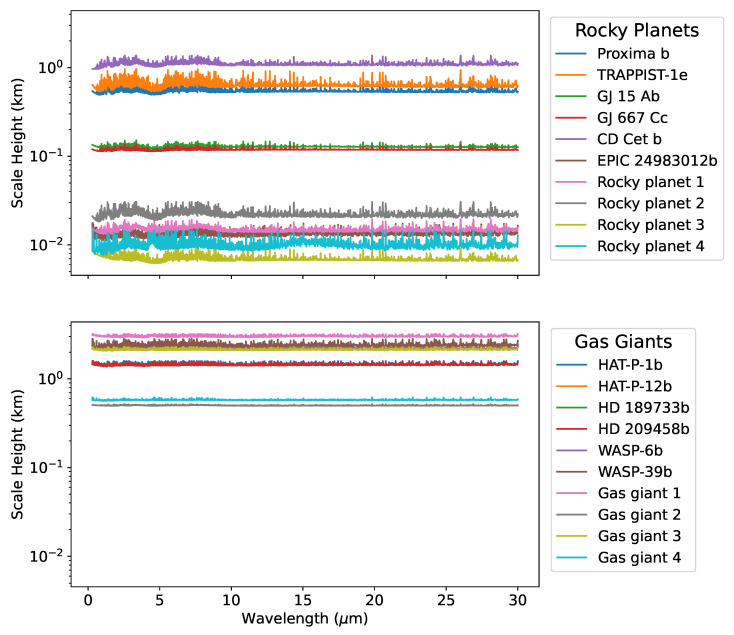
Spectra for rocky planets (Earth-like planets and super-Earths, top) and gas giant planets (Jupiter-like planets and hot Jupiters, bottom) simulated by Exo-Transmit Kempton et al. [37].

## Data Availability

The code used to generate the figures in this work is available at https://github.com/saracha413/space-djs (accessed on 24 January 2022).

## Abbreviations

The following abbreviations are used in this manuscript:

$D_{\mathrm{JS}}$	Jensen–Shannon Divergence
$D_{\mathrm{KL}}$	Kullback–Liebler Divergence
HWO	Habitable Worlds Observatory
ESI	Earth Similarity Index

## Appendix A. Noise Dependence of Jensen–Shannon Divergence

An additional advantage of using $D_{\mathrm{JS}}$ to compare distributions is that the measure scales predictably with noise. This allows us to determine how small the noise must be to wash out a signal. Suppose, for simplicity, that two signals have the same signal-to-noise ratio. This means the errors on their modal fractions, *p* and *q*, are the same. We represent this as

(A1) $$\begin{array}{cc} D_{\mathrm{JS}}\left(p+\delta_{p}\left|\right|q+\delta_{q}\right) & =\frac{1}{2}\sum \left(p+\delta_{p}\right)\log\left(\frac{p+\delta_{p}}{\frac{1}{2}\left(p+q+\delta_{p}+\delta_{q}\right)}\right)\\ \; & +\frac{1}{2}\sum \left(q+\delta_{q}\right)\log\left(\frac{q+\delta_{q}}{\frac{1}{2}\left(p+q+\delta_{p}+\delta_{q}\right)}\right) \end{array}$$

for $\delta_{p}$ and $\delta_{q}$ the noise on *p* and *q*, respectively. We may rewrite this expression so the log terms containing $\delta_{p}$ or $\delta_{q}$ take the form log(1+x) for *x* a multiple of $\delta_{p}$ and/or $\delta_{q}$. This gives

(A2) $$\begin{array}{cc} D_{\mathrm{JS}}\left(p+\delta_{p}\left|\right|q+\delta_{q}\right) & =\frac{1}{2}\sum [\left(p+\delta_{p}\right)\left(\log(p)+\log(2)\right)\\ \; & +\left(q+\delta_{q}\right)\left(\log(q)+\log(2)\right)-\left(p+q+\delta_{p}+\delta_{q}\right)\log\left(p+q\right)\\ \; & +\left(q+\delta_{q}\right)\log\left(1+\frac{\delta_{q}}{q}\right)+\left(p+\delta_{p}\right)\log\left(1+\frac{\delta_{p}}{p}\right)\\ \; & -\left(p+q+\delta_{p}+\delta_{q}\right)\log\left(1+\frac{\delta_{p}+\delta_{q}}{p+q}\right)] \end{array}$$

We may then carry out the second-order Taylor expansion, $log\left(1+x\right)\approx x-\frac{1}{2}x^{2}$ for small x. This gives

(A3) $$\begin{array}{cc} D_{\mathrm{JS}}\left(p+\delta_{p}\left|\right|q+\delta_{q}\right) & =\frac{1}{2}\sum [\left(p+\delta_{p}\right)\left(\log(p)+\log(2)\right)\\ \; & +\left(q+\delta_{q}\right)\left(\log(q)+\log(2)\right)-\left(p+q+\delta_{p}+\delta_{q}\right)\log\left(p+q\right)\\ \; & +\left(q+\delta_{q}\right)\left(\frac{\delta_{q}}{q}-\frac{\delta_{q}^{2}}{q^{2}}\right)+\left(p+\delta_{p}\right)\left(\frac{\delta_{p}}{p}-\frac{\delta_{p}^{2}}{p^{2}}\right)\\ \; & -\left(p+q+\delta_{p}+\delta_{q}\right)\left(\frac{\delta_{p}+\delta_{q}}{p+q}-\frac{{\left(\delta_{p}+\delta_{q}\right)}^{2}}{2{\left(p+q\right)}^{2}}\right)]. \end{array}$$

We perform the multiplications, again keeping only terms up to the second order in δ to find

(A4) $$\begin{array}{cc} D_{\mathrm{JS}}\left(p+\delta_{p}\left|\right|q+\delta_{q}\right) & =\frac{1}{2}\sum [\left(p+\delta_{p}\right)\left(\log(p)+\log(2)\right)\\ \; & +\left(q+\delta_{q}\right)\left(\log(q)+\log(2)\right)\\ \; & -\left(p+q+\delta_{p}+\delta_{q}\right)\log\left(p+q\right)+\frac{{\left(\delta_{p}+\delta_{q}\right)}^{2}}{2(p+q)}]. \end{array}$$

We collect the terms by their order in δ to find

(A5) $$\begin{array}{cc} D_{\mathrm{JS}}\left(p+\delta_{p}\left|\right|q+\delta_{q}\right) & =\frac{1}{2}\sum [p\log\left(\frac{p}{\frac{1}{2}\left(p+q\right)}\right)\\ \; & +q\log\left(\frac{q}{\frac{1}{2}\left(p+q\right)}\right)+\delta_{p}\log\left(\frac{p}{\frac{1}{2}\left(p+q\right)}\right)\\ \; & +\delta_{q}\log\left(\frac{q}{\frac{1}{2}\left(p+q\right)}\right)+\frac{{\left(\delta_{p}+\delta_{q}\right)}^{2}}{2(p+q)}]. \end{array}$$

The zeroth order term is simply $D_{\mathrm{JS}}\left(p||q\right)$. The change in $D_{\mathrm{JS}}$ with the addition of noise is then

(A6) $$\begin{array}{cc} D_{\mathrm{JS}}\left(p+\delta_{p}\left|\right|q+\delta_{q}\right) & =\frac{1}{2}D_{\mathrm{JS}}\left(p||q\right)\\ \; & +\frac{1}{2}\sum \left[\delta_{p}\log\left(\frac{p}{\frac{1}{2}\left(p+q\right)}\right)+\delta_{q}\log\left(\frac{q}{\frac{1}{2}\left(p+q\right)}\right)+\frac{{\left(\delta_{p}+\delta_{q}\right)}^{2}}{2(p+q)}\right]. \end{array}$$

As an example, we examine how small the noise must be in order for Earth-like planets to be distinguished both from each other and from Jupiter. We consider the two most Earth-like exoplanets in our distribution: Proxima b, a habitable zone planet with similar mass and radius to Earth, and TRAPPIST-1e, a habitable zone planet slightly smaller than Earth (suppose random noise is added to the transmission spectra for each of the planets (Earth, Proxima b, TRAPPIST-1e, and Jupiter) all drawn from the same Gaussian distribution with width σ chosen to be a particular percentage of the mean transit depth of the transmission spectrum of the planet. We would like to know how large σ must be to meet two different thresholds: one for the two Earth-like exoplanets to be indistinguishable (by $D_{\mathrm{JS}}$) from each other and another for the two Earth-like exoplanets to be indistinguishable from Jupiter. In Figure A1, we show how the $D_{\mathrm{JS}}$ (relative to Earth) varies with σ for each planet. For the noiseless spectra on the far left of the plot, all three planets are easily distinguished through $D_{\mathrm{JS}}$. As the intensity of the noise increases to the right of the plot, the $D_{\mathrm{JS}}$ of all three planets increase but at different rates. The more Earth-like the planet (lowest initial $D_{\mathrm{JS}}$), the more sensitive $D_{\mathrm{JS}}$ is to noise. This means that, with increasing noise, the three lines approach each other, causing the $D_{\mathrm{JS}}$ of the planets to be indistinguishable for large noise.

To quantify how weak the noise must be for two planets to be indistinguishably Earth-like, we arbitrarily choose two signals to be indistinguishable if their $D_{\mathrm{JS}}$ values relative to Earth are within 10% of each other. The value of σ at this threshold for distinguishing the two Earth-like planets from each other and from Jupiter is shown in gray dashed lines. For TRAPPIST-1e to be distinguishable from Proxima b, we find that the σ must be less than about 6.45% of the strength of the signals. For Proxima b to be distinguishable from Jupiter, σ must be less than about 11.73% of the signals.

We note that modeling noise for observed transmission spectra is much more complicated than the Gaussian noise method shown here as a first illustration. The noise on a transmission spectrum depends on physical parameters such as the relative size and distance of the planet and its host star, the number of observed transits, and an efficient modeling of instrument noise. In future work, we plan to use the an observation simulator such as PandExo [86] to test the efficacy of the $D_{\mathrm{JS}}$ method on JWST data, with features in Earth-like planet spectra of order 10–100 ppm [92] and especially on upcoming missions such as the HWO.

## Appendix B. Simulated Spectra

The spectra simulated in this work are shown for reference in Figure A2.
